# Anaplastic Variant of Classical Seminoma of the Testis: Northern Israel Oncology Center Experience and Brief Review of Literature

**DOI:** 10.5041/RMMJ.10140

**Published:** 2014-01-21

**Authors:** Moshe E. Stein, Jamal Zidan, Tomer Charas, Karen Drumea, Rahamim Ben-Yosef

**Affiliations:** 1 Northern Israel Oncology Center, Rambam Health Care Campus, and Faculty of Medicine, Technion-Israel Institute of Technology, Haifa, Israel and; 2 Institute of Oncology, Ziv Medical Center, Safed, Israel

**Keywords:** Anaplastic seminoma, early stage, good prognosis, radiotherapy

## Abstract

**Objectives:**

There are only sporadic reports on the clinical behavior and appropriate treatment of anaplastic seminoma. This retrospective study summarizes our experience with the anaplastic variant of classical (typical) seminoma.

**Methods:**

Between 1986 and 2006, seven anaplastic seminoma patients were staged and treated at the Northern Israel Oncology Center. Staging procedures included meticulous physical and neurological examinations, complete blood count, full biochemistry profile, specific tumor markers, testicular ultrasound, and other radiological measures. All patients underwent inguinal orchiectomy and were staged properly. Six patients had stage I disease, and one patient had stage IIA disease. Patients were irradiated with doses ranging from 2,500 to 3,000 cGy, and the stage IIA patient received an additional 1,000 cGy boost to radiographically involved lymph nodes.

**Results:**

After a mean follow-up of 11 years, six patients are alive with no evidence of disease. One patient died due to an unknown, non-oncological, cause, unrelated to his previous testicular tumor, while in complete remission.

**Conclusions:**

Despite the low patient numbers and the retrospective nature of our study, it can be concluded that radiotherapy treatment for early-stage anaplastic seminoma patients might achieve the same excellent survival as for classical seminoma. However, the general consensus achieved through large-scale studies suggests that active surveillance should be offered to all stage I seminoma patients, regardless of the pathologic variant.

## INTRODUCTION

At the present time, testicular seminomas are commonly categorized into classical seminoma (CS), and into the spermatocytic and anaplastic variants of CS. Mostofi^1^ established the diagnosis of anaplastic seminoma (AS) in tumors with overall morphologic features of seminoma but with more than three mitotic figures per high-powered field (HPF), cellular irregularity, no fibrovascular septae, few lymphocytes, focal necrosis, and pleomorphic cells with non-clear cytoplasm. On the other hand, Von Hochstetter^2^ suggested that, if mitotic activity continues to be used in separating AS from CS, the critical threshold should be elevated to six mitoses per HPF. According to these criteria, AS constitutes 5%–15% of testicular seminomas.^3^

There are conflicting reports about the clinical behavior of AS. While Kademian et al.^4^ and Mostofi and Price^5^ demonstrated an aggressive clinical pattern, other authors^6^,^7^ demonstrated no difference in clinical behavior between AS and CS and suggested that treatment should be the same, stage for stage. There are very few studies and limited information about AS in recent years. The goal of our study was a retrospective screening of seven AS patients staged and treated with radiotherapy post-orchiectomy at the Northern Israel Oncology Center (NIOC) in the years 1986–2006.

## PATIENTS AND METHODS

From 1971 to 2010, 112 stage I and 26 IIA seminoma patients were referred to the NIOC for meticulous post-orchiectomy staging and radiation therapy. Seven patients demonstrated the Mostofi microscopic criteria for AS.^1^ All patients underwent clinical examination, full hematological and biochemistry profile, specific tumor markers (alpha fetoprotein (AFP), beta-subunit of human chorionic gonadotropin (B-HCG), lactate dehydrogenase (LDH)), and whole-body computerized (CT) scan. Follow-up was provided through regular follow-up visits and a search of the Interior Ministry’s electronic data.

Staging resulted in six stage I (disease confined to testis) and one stage IIA (para-aortic lymphadenopathy 2 cm in size) patients. Initially, radiotherapy was applied with 6–8 megavoltage photons following 2-dimensional planning with anatomic bony landmarks and posterior/anterior fields. Since 1990, 3-D conformal CT-based planning was implemented.^8^ Four stage IA patients were irradiated with the “hockey stick” method (para-aortic lymph nodes and ipsilateral iliac lymph nodes) and three patients with the “inverted-Y” method (para-aortic and bilateral pelvic lymph nodes), with a total dose ranging between 2,500 and 3,000 cGy, daily fractions of 125–200 cGy, five times weekly. The stage IIA patient was additionally boosted to the radiographically demonstrable para-aortic tumor bulk with 1,000 cGy (daily fraction of 125 cGy). Two patients received additional radiotherapy to the inguinal area, due to adverse factors which might predict relapse (one patient: perineural and lymphogenic invasion, spermatic cord involvement; one patient: rete testis invasion). Boost was given with 6 megavoltage photons and CO-60 to a total dose of 2,500 cGy and 125 cGy daily fractions, respectively.

## RESULTS

Mean age of patients was 33 years (range, 27–43 years). Three were Jews and four were Arabs. Only one patient was not born in Israel (Russian-born). An etiological factor (cryptorchidism) was evaluated in one patient. The tumor was confined to the right side in four patients. Symptoms included testicular enlargement and/or mass, pain in three patients, and a hydrocele in one patient. Mean duration of symptoms was 3 months (range, 1–8 months). With a mean follow-up of 11 years (range, 2–24 years) calculated from surgical procedure to last follow-up, five patients are alive with no evidence of disease, chronic severe side effects, or second primary. One patient was lost to follow-up, and one died due to an unknown cause unrelated to his primary disease 12 years after diagnosis.

## DISCUSSION

Between 5% and 15% of all testicular seminomas are histologically classified as AS.^3^ However, due to the low number of AS patients mentioned in scientific studies and the retrospective nature of these studies, it is difficult to determine whether the anaplastic differentiation predicts bad prognosis, like other solid tumors with anaplastic biology.^3^ Kademian et al.,^4^ in their 1977 study, and Bobba et al.,^9^ in their 1988 study, demonstrated a worse prognosis and higher relapse rate compared to CS. Percarpio et al.,^7^ who summarized the treatment results of 77 AS patients in three large medical centers and after a follow-up of 28 years, found the same excellent survival rates in AS and CS patients in early stage following orchiectomy and radiation therapy. They concluded that the treatment decision in AS patients should be based on stage and generally accepted adverse factors like size, lympho-vascular invasion, and rete testis involvement. Cockburn et al.^3^ came to the same decision (25 patients), as did Maier and Sulak^6^ (39 patients).

Hence, the sub-classification of seminoma into well-differentiated and undifferentiated for purposes of treatment and prognosis may be doubted on the basis of the Memorial Sloan–Kettering Cancer Center (MSKCC) experience.^3^ Ultrastructural studies and electron microscopic appearance^2^,^10^,^11^ have failed to reveal significant differences between AS and CS. Moreover, the poor prognosis of AS in the past could be related to understaging in the pre-CT era and misdiagnosis of aggressive testicular lymphoma and embryonal carcinoma based on light microscopy alone without histochemistry studies.^2^,^3^,^12^,^13^

Summarizing our and world-wide accumulating experience and current policy for stage I seminoma, as emphasized by Schmoll et al.,^14^ Albers et al.,^15^ and de Wit and Bosl,^16^ it is agreed that standard management has shifted largely to active surveillance or a single cycle of carboplatin with area under the curve (AUC 7) for low-risk patients. Concerning radiation therapy, only high-risk factors, such as tumor size larger than 4 cm, rete testis involvement, and vascular invasion, should be treated with radiation therapy (total dose 20–25 Gy) to the para-aortic field alone, omitting the pelvic fields. Radiotherapy planning should be 3-D conformal CT-based or intensity-modulated radiotherapy, aiming to reduce the dose to active bone marrow and radio-sensitive abdominal organs, hence reducing potential late toxicity and second malignancies.

## CONCLUSION

Treatment of anaplastic seminoma should be the same as for classical seminoma, stage for stage. Currently, surveillance policy should be implemented for all stage I seminoma, regardless of the pathologic variant.

## Abbreviations:

AFP: alpha fetoprotein;
AS: anaplastic seminoma;
B-HCG: beta-subunit of human chorionic gonadotropin;
CS: classical (typical) seminoma;
CT: whole-body computerized scan;
HPF: high-powered field;
LDH: lactate dehydrogenase;
MSKCC: Memorial Sloan–Kettering Cancer Center;
NIOC: Northern Israel Oncology Center.
